# Shared understanding of resilient practices in the context of inpatient suicide prevention: a narrative synthesis

**DOI:** 10.1186/s12913-022-08282-x

**Published:** 2022-07-29

**Authors:** Siv Hilde Berg, Kristine Rørtveit, Fredrik A. Walby, Karina Aase

**Affiliations:** 1 Centre for Resilience in Healthcare, Faculty of Health Sciences, University of Stavanger, Kjell Arholmsgate 43, N-4036 Stavanger, Norway; 2 Clinics of Adult Mental Health Care, Stavanger University Hospital, P.O. Box 8100, N-4068 Stavanger, Norway; 3 Milieu Therapy and Mental Health Nursing Research Group, Clinics of Adult Mental Health Care, Stavanger University Hospital, P.O. Box 8100, N-4068 Stavanger, Norway; 4 Life Phenomena and Caring Research Group, Department of Caring and Ethics, Faculty of Health Sciences, University of Stavanger, N-4036 Stavanger, Norway; 5 National Centre for Suicide Research and Prevention, Faculty of Medicine, University of Oslo, Sognsvannsveien 21, Building 12, N-0320 Oslo, Norway

**Keywords:** Patient safety, Inpatient suicide, Suicide prevention, Adaptation, Mental health, Resilient healthcare, Safety science, Complexity

## Abstract

**Background:**

The prevailing patient safety strategies in suicide prevention are suicide risk assessments and retrospective reviews, with emphasis on minimising risk and preventing adverse events. Resilient healthcare focuses on how everyday clinical practice succeeds and emphasises learning from practice, not from adverse events. Yet, little is known about resilient practices for suicidal inpatients. The aim of the study is to draw upon the perspectives of patients and healthcare professionals to inform the conceptual development of resilient practices in inpatient suicide prevention.

**Methods:**

A narrative synthesis was conducted of findings across patients and healthcare professionals derived from a qualitative case study based on interviews with patients and healthcare professionals in addition to a systematic literature review.

**Results:**

Three sub-themes categorise resilient practices for healthcare professionals and for patients hospitalised with suicidal behaviour: 1) interactions capturing non-verbal cues; 2) protection through dignity and watchfulness; and 3) personalised approaches to alleviate emotional pressure. The main theme, the establishment of relationships of trust in resilient practices for patients in suicidal crisis, is the foundation of their communication and caring.

**Conclusion:**

Clinical practice for patients hospitalised with suicidal behaviour has characteristics of complex adaptive systems in terms of dynamic interactions, decision-making under uncertainty, tensions between goals solved through trade-offs, and adaptations to patient variability and interpersonal needs. To improve the safety of patients hospitalised with suicidal behaviour, variability in clinical practice should be embraced.

**Trial registration:**

https://doi.org/10.1136/bmjopen-2016-012874

## Introduction 

Our understanding of patient safety for patients hospitalised during a suicidal crisis is limited [1]. In a systematic review of the literature, Thibaut et al. found [2] few peer-reviewed empirical studies on patient safety and suicidal behaviour. The literature drawing on the interdisciplinary field of safety science in suicide prevention is particularly thin. Safety science has provided us with the key message that different models affect our understanding of adverse events and thereby have a vast impact on learning and to where we direct our safety measures [3–5].

According to Hollnagel, the traditional view of safety, referred to here as safety-I is defined by the absence of accidents and incidents [6]. Consequently, the focus of safety management and research has been on learning about unsafe systems while minimising the number of adverse events [7]. Methods such as root cause analysis, incident reporting and checklists to reduce healthcare professionals’ (HCPs’) errors are examples of the traditional approach to patient safety [7, 8]. Likewise, in a scoping review of patient safety strategies in psychiatry, Svensson found that these measures are mainly intended to prevent adverse events by focusing on failure and ensuring compliance to procedures [9]. Across countries, clinical practice guidelines on suicide prevention include restricting lethal means, assessing evidence-based suicide risk factors and suicidal intent along with recommended treatment and post-intervention recommendation [10]. Nevertheless, while the clinical field is moving in the direction of a systematic suicide management system [11–13], patient safety strategies in suicide prevention have been criticised as excessively focused on harm reduction (e.g. suicide risk assessment) [14–16].

Retrospective reviews of suicide incidents have focused on deficiencies in the interface between healthcare professionals (HCPs) and patients [14, 17, 18]. In hindsight, suicide investigations have often concluded that an improved risk assessment could have led to a different outcome [14, 17, 18]. Nonetheless, suicidal outcomes cannot be predicted at the individual level [19–21]. The fallacies of perceiving HCPs in the sharp end of the system as the main causes of the patient suicide are abundant; HCPs are the secondary victims of patient suicides; learning outcomes at the system level are limited; and the complexity of making the decisions regarding suicide risk, and the flexibility required for HCPs to adapt treatment to patients is ignored [14, 17, 18].

A safety-I approach narrows the gap between what HCPs do in practice and their procedures for that practice [6]. Patient safety in psychiatry has emphasised improving staff compliance to safety-I procedures [9]. At the same time, there is no single means of preventing inpatient suicides, and practice is characterised by uncertainty and complexity. The lack of a solution relates to the aetiological heterogeneity of suicidal patients, and the need to understand and approach each patient differently [22]. Uncertainty is also related to the ethical challenges of assigning suicidal patients to a control non-treatment condition to determine whether specific interventions, such as constant observation, have a preventive effect [23]. As such, no study has examined whether being under observation reduces the number of suicide attempt or deaths [24]. Clinical decision-making regarding suicide risk involves a high degree of uncertainty. Despite considerable research, instruments used to assign patients to high-risk groups do not enable HCPs to predict which of those patients will die by suicide [19, 25, 26]. There are therefore many limitations to the safety-I approach in the face of healthcare practices.

The field of resilient healthcare (RHC) has sought insight into healthcare practices and the ways in which everyday clinical practice succeeds [27–29]. Hollnagel [6] has defined safety-II as ‘the ability to succeed under expected and unexpected conditions alike, so that the number of intended and acceptable outcomes (in other words, everyday activities) is as high as possible’ (Hollnagel, 2014, p. 134). RHC draws on theories from safety science in which healthcare is recognised as a complex adaptive system (CAS) [27, 28]. Wiig et al. [30] describe resilience in healthcare as ‘…the capacity to adapt to challenges and changes at different system levels to maintain high-quality care’ (p. 6). Adaptation is thus seen as the central tenet of RHC and might come in many forms and at multiple levels, for example as HCPs’ capacity to adjust their reasoning and clinical measures based on patient conditions and/or work-related conditions.

Mental health services have been conceptualised as a CAS by Ellis, Churruca and Braithwaite [31], in which patients and HCPs interact across care levels, and constantly adjust practice to the patient, caregivers, policies, research and standardised and evidence-based treatment. Although most RHC studies have used HCPs as the main source of data on healthcare resilience, patients and family may also be sources [32, 33]. Quinlivan et al. [34] emphasise the importance of patient perspectives when applying an RHC approach to suicide prevention to better grasp the patient vulnerabilities and the individual differences specific to this field. However, patients have not been used extensively as sources of knowledge in RHC [35, 36]. Furthermore, few empirical studies have adopted RHC perspectives to suicide prevention [37–39], and none have incorporated patients’ perspectives. This synthesis of an in-depth qualitative case study applying RHC to suicide prevention can fill this gap. Therefore, the aim of this study was to draw upon the perspectives of patients and healthcare professionals to inform the conceptual development of resilient practices in inpatient suicide prevention.

## Methods

A narrative synthesis was conducted of findings across two embedded units of analysis: the experiences of patients and the experiences of HCPs (Table 1) [40]. The original data consisted of three samples collected through a multi-method case study approach [40] consisting of a systematic review of 20 peer-reviewed articles published between 1999 and 2016 on patient experiences of being hospitalised in a psychiatric ward or facility during a suicidal crisis (sample 1) [41]; individual interviews with 18 patients hospitalised during a suicidal crisis (sample 2) [42]; and focus group and individual interviews with 35 HCPs responsible for the care of suicidal patients (sample 3) [43]. The methods of the case study are described in a published study protocol [44] and the first author’s PhD thesis [45]. Interview guides have been previously published, see supplemental file 2 in Berg et al. 2020a [46] and additional file 1 in Berg et al. 2020b [47].

**Table 1:** Overview of the three samples included in the case study synthesis adapted from Berg,2020 [45]

Embedded unit of analysis	Patients’ experiences		Healthcare professionals’ experiences	
Sample	Sample 1	Sample 2	Sample 3	
Data collection methods	Systematic review	Individual interviews	Focus group interviews	Individual interviews
Participants/ material	20 peer-reviewed articles, participants in total (*n* = 311)	18 patients	35 HCPs (focus groups (*n* = 25), individual interviews (*n* = 18), of which 8 participated in both)	
Analysis methods	Thematic analysis	Qualitative content analysis Phenomenological hermeneutical approach	Qualitative content analysis Phenomenological hermeneutical approach	

The literature review (sample 1) followed a systematic approach according to the PRISMA guidelines (Preferred Reporting Items for Systematic Reviews and Meta-Analyses [48]. Systematic searches were conducted in the MEDLINE, Academic Search Premier, CINAHL, SOCINDEX and PsycINFO databases, identifying 20 qualitative studies on suicidal patients and their psychiatric in-patient care experiences. These studies were systematically reviewed according to PRISMA, subjected to quality appraisals, and synthesized by using thematic analysis [49, 50]. The included studies had a total of 311 patients (see Table 2).

All individual interviews followed a semi-structured interview guide and were conducted by SHB face to face in a room close to the hospital ward. Focus groups were conducted with a moderator (SHB/KR) and a co-moderator (SHB/MA). Sample 3 followed a sequential triangulation, with the first data set (focus groups) informing the nature of the second (individual interviews) [51]. All interviews were audio-recorded, transcribed verbatim, coded and followed the systematic qualitative analysis methods of thematic analysis [50] and content analysis [52], with a phenomenological hermeneutical approach [53].

The setting for the empirical studies (sample 2, sample 3) was a university hospital in Norway. We used purposeful sampling to recruit suicidal patients and HCPs working in open or locked wards in specialised mental healthcare facilities for adults. Participants were recruited from nine wards. Locked wards specialised in affective disorders (*n* = 2) or acute care (*n* = 2) while open wards specialised in rehabilitation (*n* = 3) or short-time stabilisation during a crisis (*n* = 2). Fifty-three individuals (patients and HCPs) participated in the empirical studies. All patient participants had active suicidal ideation during inpatient care, nine had recently attempted suicide prior to admission to mental healthcare, see Table 2 (sample 1 and 2).

**Table 2:** Sample characteristics

	**Sample 1**	**Sample 2**
Gender	NA	Male (*n* = 7), female (*n* = 11)
Age	16- 63 years	18–57 years
Number of hospitalisations	NA	First time (*n* = 4) 2–22 (*n* = 11) 50 > (*n* = 3)
Diagnoses	Affective disorders most frequently diagnose (major depression most prevalent), followed by schizophrenia spectrum diagnoses and personality disorders	Affective disorder as main diagnosis/ comorbid diagnosis (*n* = 18) Psychotic episode during admission (*n* = 4) Comorbid diagnoses, including mental and behavioural disorders due to alcohol use, depression, posttraumatic stress disorder and attention deficit/hyperactivity disorder
Suicidal behavior	Active suicidal ideations during inpatient care (*n* = 311) The majority had recently attempted suicide	Active suicidal ideations during inpatient care (*n* = 18), Recently attempted suicide (*n* = 9)

The HCPs sample included both novices and experienced participants from all professional groups, see Table 3 (sample 3).

**Table 3:** Sample Characteristics

Sample 3	
Gender	Male (*n* = 7)
	Female (*n* = 28)
Work setting	Locked wards (n = 14) Open wards (*n* = 21)
Professional groups	Nurses (*n* = 22), Medical doctors (*n* = 7) Psychologists (*n* = 6)

Two patients who were invited to participate declined because of their mental state. No HCPs refused to participate. None dropped out. Five patients had follow-up interview within a week of the first interview, which followed the same interview guide.

### The Norwegian suicide context

Preventing suicides during inpatient care is a high priority for patient safety in mental health. In Norway, the national guidelines for the prevention of suicides in mental healthcare [54] outline practices that managers and HCPs in specialised mental healthcare are required to follow. In addition, checklists have been used in the management of suicide risk [55]. Suicide rates have remained relatively stable in Norway, with approximately 12.1 suicides per 100 000 inhabitants in 2020 [56], which is slightly higher than the global suicide rate (11.4 per 100 000 inhabitants in 2020 [57]. Approximately 650 individuals die from suicide in Norway each year [58], and there are approximately 3500–7500 suicide attempts annually [59]. Walby et al. [60] found that 67% of the individuals in Norway who died by suicide within a year of last contact with mental health services had been hospitalised at least once in the year preceding their death. On average, 25 suicides (SD = 4,9) occurred during inpatient care each year in Norway in the period 2008–2015. This is a relatively low proportion (13%) of the total number of suicides conducted by patients receiving specialised mental healthcare services, meaning that most suicides occur in the outpatient care [60].

### Ethical considerations 

The case study was approved by the Western Regional Ethics Committee of Norway (REC 2016/34). All participants provided voluntary informed and written consent. Patients were interviewed during hospitalisation and all of them were under the care of specialised HCPs. None of the researchers had a role as a clinician or manager in any of the seven units included in the study or had prior established relationships with the participants. Participants were informed of the rationale for the research: to get insight into safe clinical practices for suicidal patients.

### Analysis 

A narrative synthesis was conducted across methods and samples to find common themes reflecting both patients’ and HCPs’ perspectives, using the four stages of data synthesis as proposed by Whittemore and Knafl [40]. The narrative synthesis was a re-analysis of the work presented in the first author’s PhD thesis [45]. In the first stage, the primary sources were divided into two subgroups: patients and HCPs. In the second stage, the main findings related to perspectives on resilient practices were extracted from the primary sources and coded inductively. In the third stage, tables were used to display the codes to visualise patterns and relationship within and across primary data. Categories relating to both the patients’ and the HCPs’ perspectives were constructed by compiling codes with shared meaning. In the fourth stage, data were organised into meaningful patterns with themes and sub-themes under an overarching main theme.

## Results 

This narrative synthesis synthesised patients’ experiences and the HCPs’ experiences in the context of inpatient suicide prevention. The main theme, relationships of trust in resilient practices for patients in suicidal crisis, is the foundation of their communication and caring. The main theme was described by three sub-themes which illuminate three sets of relationships: interactions capturing non-verbal cues, protection through dignity and watchfulness, and personalised approaches to alleviate emotional pressure. Table 4 depicts the main theme, the three sub-themes and categories related to the patient and HCPs perspective and how the three samples form the three themes.

**Table 4:** Overview of the narrative synthesis

Main theme	Relationships of trust in resilient practices for patients in suicidal crisis		
Sub-themes	**Interactions capturing non-verbal cues**	**Protection through dignity and watchfulness**	**Personalised approaches to alleviate emotional pressure**
Patients’ perspective	Struggle to communicate suicidal ideations [2]	Safe balance among multiple needs for protection [1,2]	Regaining a sense of control through coping with symptoms and difficulties [1,2]
HCPs’ perspective	Attending to multiple sources of information to make sense of suicidal behaviour [3]	Adjusting protection of the patient through dynamic trade-offs between under- and over-protection [3]	Targeting underlying issues by creating individual clinical pathways [3]

**Sources**

[1] = Sample 1, systematic review of patient experiences

[2] = Sample 2, interview study with patients

[3] = Sample 3, interview study with HCPs

The main theme, relationships of trust in resilient practices for patients in suicidal crisis reflected that resilient practices depended on trust between patients and HCPs, and that HCPs need alliances to establish trusting relationships with the patients. Trust is a precondition for resilient practices to adapt protection, treatment, and care to the needs of suicidal patients.

Suicidal patients experienced the relationship to the HCPs as essential for a feeling of safety during inpatient care. They felt safe when they met someone who cared, validated their feelings and respected them as human beings. Trusting and familiar relationships with HCPs gave them predictability in terms of how their suicidal behaviour would be understood and treated. Trust is relevant to findings involving patients in vulnerable situations: being externally controlled, talking about suicide and relieving emotional pressure.

HCPs experienced personal uncertainty in working with patients in suicidal crisis. They feared losing a patient to suicide while under their care, and they feared being held responsible by the health authorities and the hospital management. HCPs noted that their colleagues’ trust and support were necessary to manage uncertainty in clinical practice. HCPs formed and adapted their informal networks in response to the lack of formal support systems. Without support that could help HCPs cope with the uncertainty of working with suicidal patients, HCPs resorted to fear-based defensive practices to avoid blame and responsibility, which took time and focus away from the personalised activities with patients. However, social support and clinical supervision helped to counteract the impact of personal uncertainty in the patient contact.

As such, resilient practices did not merely depend on strengthening the HCPs’ ability to establish trusting relationships with the patients, but also on ensuring that HCPs had reliable support systems.

### Interactions capturing non-verbal cues 

The resilient practice interactions capturing non-verbal cues described the interaction between the patient and the HCPs in the detection of suicidal crisis. Suicidal crisis was apparent not only in what patients did and did not say, but also in their body language and affect, as exemplified in Table 5.

**Table 5:** Exemplifying quotes related to interactions capturing non-verbal cues

Patients	*I have to be looked after when it gets serious enough. I don’t say anything when I’m suicidal. I get very fixated and cunning. I’m not thinking about anything but dying. That’s why they know that they have to keep me protected. I am just doing it [suicide attempt]. When I got hospitalised I was just extremely depressed and started losing the ability to talk and the ability to express myself. I don’t think I realised I was hospitalised at first. I was completely confused…I got through this crisis, because they know me, and that is why I think it is important to be admitted to the same ward. They have seen it in the change of my mental state, the things I say and don’t say, my facial expressions. They have read me when I get truly, truly silent; then I am ill, and they watch me extra closely. I have survived because they have watched me like hawks. They have given me my personal freedom, but not too much* (Female, bipolar disorder, locked ward, sample 2)
Healthcare Professionals	*Suddenly there may be a minor sentence such as ‘it is just for the best that the father has the child custody rights’. At the time I may think it is weird, feel a sense of unease and go back into the patent room and ask, «what did you mean by that?»…But a lot of times we don’t catch it at all, because you’re on to the next patient long before you really get to leave their patient room. Sometimes, when I’m at home I can feel my heart beating. Those are the times I’ve called back and discussed it with the team…I can feel it just by being with them, and many times, especially if I know the patient, I can feel it before they can express it with words. She can tell me to leave and say everything is fine, and I will tell her that I feel I don’t want to leave you; I will stay. And often, after a while, she can explain she had suicidal plans at that moment* (Female nurse, 24 years of experience, open rehabilitation ward, sample 3)

Many of the patients expressed a struggle to communicate suicidal ideations during suicide risk assessment and acute suicidal crisis in the ward. They also experienced being recognised by HCPs who were attentive to their body language and to changes in their mental state. Patients noted that when their HCPs took a genuine personal interest in them, they were less likely to attempt suicide during inpatient care (sample 2).

HCPs attended to multiple sources of information to make sense of suicidal behaviour including the non-verbal cues. The more experienced HCPs claimed that the formal procedures for assessing risk were not helpful during risk assessment, as the list did not take the context-sensitive and non-verbal behaviour into account. They improved their understanding by establishing relational contact with the patient and triangulating several sources of information, including the patients’ verbal and non-verbal cues, mental state, diagnosis and their own ‘gut feeling’. Expertise increased the complexity and the number of information sources that were considered to clarify the picture. Furthermore, HCPs improved their situational awareness of the patients’ suicide risk by seeking other perspectives, which involved reflecting with the patient, bringing other HCPs into conversations with the patient and discussing the case with more experienced colleagues and with doctors on call (sample 3).

Interactions capturing non-verbal cues were grounded in relationships of trust. Patients described a trusted HCPs as someone who connected with them and understood their verbal and non-verbal cues of suicidal crisis (sample 2). When trusted HCPs were unavailable, patients in suicidal deterioration felt unsafe and vulnerable (sample 2). HCPs used experience and emotionally based competence to anticipate suicidal acts, and they perceived this process was strengthened by developing bonds of trust with the patient (sample 3).

### Protection through dignity and watchfulness

The resilient practice protection through dignity and watchfulness described the physical protection of patients with suicidal behaviour as a relational and interacting practice, depending on adaptations to ensure that each patient was physically protected, yet connected, as exemplified in Table 6.

**Table 6:** Exemplifying related to protection through dignity and watchfulness

Patients	*Intermittent observation can make me feel safe, but it depends on how suicidal I am. It does not work if I just want to die and don’t want any help, but it does work when I just don’t want to live, but am not driven by suicidal impulses… But they need to interact with me somehow and ask me how I am doing…. I remember one time I had an observation every 15 min. When they opened the door and looked in, I just felt like they were constantly saying ‘yes, she’s still alive’, and then they left again. Then it loses its purpose because it was okay that they saw I was alive, but no one asked how I was doing. And that is the clue, and they cannot merely act as prison guards* (Female, emotional unstable personality disorder, open rehabilitation ward, sample 2)
Healthcare Professionals	*Sometimes, it’s not appropriate to just look in at them. For example, we recently had a man hospitalised who had observations at five-minute intervals. I saw that he had a desire to talk, but then it almost becomes a rejection when you go out again, just to come back after five minutes and go out again. It’s like ‘I just need to check that you’re still alive’. That’s not a worthwhile thing to do. I always try to get them out of the room, so that it becomes less artificial, and I can focus my attention on them. Then the relationship becomes more equal, instead of one-sided.. I bring them out in the living room in the locked unit, but I keep an eye on them. It’s about normalising the situation, because it is not normal to have someone watching you every five minutes. They also need to experience that they do not act on their suicidal impulses* (Male nurse, 1 year of experience, locked ward, sample 3)

Suicidal patients expressed a need for a safe balance among multiple needs for protection. Findings reflected that suicidal patients needed to be physically protected from death during suicidal crisis while feeling connected with the HCPs during observation. Lack of connection during observation made some patients feel unsafe and thereby intensified their suicidal behaviour (sample 1).

Furthermore, patients’ need for protection during suicidal crisis changed in the course of hospitalisation and depended on a) their ability to establish relations with their HCPs and verbalise their needs; b) their need to withdraw from and master stressors in the outside world; c) their need for closeness to or distance from the HCPs; and d) their need for external control (e.g., locked doors) or internal control (e.g,. impulse control). Thus, resilient practices were experienced when protection of the individual balanced his or her multiple needs (sample 2).

HCPs were adjusting protection of the patient through dynamic trade-offs between under- and over-protection. While the formal procedure described observation as either constant or intermittent (5-, 10-, 30-min) intervals of observation, HCPs were constantly adjusting the observation practice towards the patient. Instead of watching the patients from a distance, they engaged with them, treated them with dignity, but kept an eye on them. Furthermore, HCPs were constantly balancing the physical protection of the patient from suicidal impulses with the risk of over-protection while increasing the patient’s sense of independence at the risk of under-protection. Thus, safe levels of protection were trade-offs between short- and long-term goals in care (sample 3).

Protection through dignity and watchfulness was rooted in relationships of trust. The link between feeling safe and being safe was vital for suicidal patients; their physical safety could not be guaranteed if they felt vulnerable (sample 1). Lack of support from HCPs when patients were under constant observation made patients feel alienated and objectified, their suicidality worsened, and it supported their perception of worthlessness and the sense that no one cared about them (sample 1). Trust that HCPs were acting in the patients’ best interest was a prerequisite for patients’ feelings of safety when they were under external control (sample 2). Likewise, HCP adapted their approaches to protection to ensure patients understood they were acting in their best interest through explaining why doors were locked, and why they had to give away some personal belongings (sample 2) and treating patients with dignity during observation (sample 3).

### Personalised approaches to alleviate emotional pressure 

The resilient practice personalised approaches to alleviate emotional pressure described the adaptation of treatment and safety procedures that are made to meet individual patients’ needs. Patients experienced less emotional pressure and their suicidal impulses diminished as a result (Table 7).

**Table 7:** Exemplifying quotes related to personalised approaches to alleviate emotional pressure

Patients	*To feel safe from myself, I needed to get out of that psychosis where I believed that I was bound to kill myself because I had let everything and everyone down. Because I did not truly want to kill myself…I lost my sense of self, my motor control, my sight and my concentration during the psychosis. I thought this was what my life had become…* *It was hopeless when I was very psychotic. I could not attend to any of what they [HCP] said, and I didn’t say anything to them. I was afraid to be locked inside forever. The most important thing for me right then was that they let my parents visit with me on the seclusion unit daily. That’s when I realised someone loved me. But also getting the medication, and the seclusion were important for me in the first phase, I guess* (Male, psychotic symptoms, locked ward, sample 2)
Healthcare Professionals	*If you talk with the patient about suicidality in every conversation, then you can tick off the list that you have done it and it calms the therapist down. But I doubt that it will calm the patient. Suicidality is a symptom the patient has. Previously we would transfer a patient to the locked wards if they were suicidal, but now we explore their grief and understand phenomenologically what lies behind the suicidality for each individual…Through gaining insight, the patient finds other ways to express their emotions… We do not simply detach ourselves from the suicidal behaviour or medicate it away… This therapy [interpersonal therapy] is always about individual processes. They get the same structural parts of the treatment, but what they need is very individual* (Male psychologist, 15 years of experience, locked ward, sample 3)

Patients emphasised that regaining a sense of control through coping with symptoms and difficulties was vital when they were having a suicidal crisis. Perception of control through gaining insight, coping with underlying difficulties and symptoms, and being prepared for discharge were essential for suicidal patients’ feeling of safety during inpatient care (sample 1).

Personalised approaches to treatment addressed patients’ underlying mental health issues and stressors. This kind of treatment relieved their emotional pressure, whether it involved medications and physical protection to reduce psychotic symptoms or resolving financial issues to instill hope and a sense of relief from depression. These strategies helped suicidal patients feel safe from suicidal impulses through strengthening their sense of internal control. Talking about suicide also alleviated patients’ individual stressors, symptoms, or emotional pain. When conversations about suicide opened with standard questions and answers about suicide risk, they lost their therapeutic benefit and could evoke feelings of shame and hopelessness. Patients achieved a sense of control in a variety of ways. In the acute phases of their suicidal crisis, some patients wanted external control over their suicidal impulses because physical protection convinced them that they could not harm themselves (sample 2).

HCPs targeted underlying issues by creating individual clinical pathways for suicidal patients. HCPs considered individualised approaches to be important in conversations about suicidal ideations; however, they experienced competing goals between documenting risk and understanding patients as individuals in practice (sample 3).

Personalised approaches to alleviating emotional pressure are rooted in relationships of trust. Seeking help and talking about suicide place a suicidal patient in a highly vulnerable position. If they could not trust their HCPs, some patients withdrew from talking about suicidal ideations and stopped seeking help (sample 2). Likewise, HCPs perceived trusted relationships as a condition for honest responses in suicide risk assessment and described setting aside checklists and forms to prioritise making a trusting bond, a safe atmosphere and engaging with the patients in a dialogue when talking about suicide (sample 3).

## Discussion 

We identified three sub-themes categorising resilient practices for patients hospitalised with suicidal behaviour and HCPs: interactions capturing non-verbal cues, protection through dignity and watchfulness, and personalised approaches to alleviate emotional pressure. All three are grounded in relationships of trust in resilient practices for patients in suicidal crisis. These resilient practices outline a safety-II perspective in inpatient care of suicidal patients, which is based on culturally situated knowledge which cannot be generalised to all inpatient settings [61]. However, theoretical conceptualisation can be made regarding the understanding of safety for the suicidal patient, from a resilience perspective [61]. Above all, the perspective of resilience provides insight into the system complexity of suicide prevention, and how patients and HCPs cope with challenges and changes [28, 62].

### The complexity of clinical practice for patients hospitalised with suicidal behaviour 

The shared understanding of resilient practices offers insight into clinical practice for patients hospitalised with suicidal behaviour. This clinical practice has the characteristics of a CAS [27, 28, 63, 64], which has been previously presented in the first author’s PhD thesis [45].

Interactions capturing non-verbal cues of suicidal inpatients reflect uncertainty in clinical decision-making. The findings correspond with previous studies suggesting that caring for suicidal patients involves uncertainty [65–67] and expand the literature by showing that making sense of suicidal behaviour requires the comprehension of information ‘beyond the spoken word’, using intuition along with other sources of information (e.g. diagnosis, mental state, medical journal, individual risk factors) [68, 69]. To attempt anticipating suicide, and to adapt and respond to the suicidal crisis of patients in the wards, HCPs apply multiple sensemaking strategies to improve their situational awareness [70, 71]. The complexity and ambiguity of the cues involved in making sense of suicidal behaviour highlight that it is difficult to standardise screening questions or limit a set of cues to look for (proximal risk factors) in the setting of inpatient suicide prevention with high specificity [72]. Furthermore, suicide prediction models are imprecise [21], the evidence does not support the use of risk scales in suicide risk assessment [20, 73], and there is no support for the use of clinical intuition as the sole source of suicide prediction, which leaves HCPs with limited support for their clinical decision making. This study supports that ‘work as done’ of suicide risk assessments involves multiple strategies to make sense of uncertainty, which is a characteristic of decision making in complex high-risk and ambiguous work settings [70, 74–76].

Protection through dignity and watchfulness, and personalised approaches to alleviate emotional pressure emphasise that there is no one-size-fits-all approach to the protection and treatment of suicidal patients. Instead, practice relies on interpersonal and context-sensitive adaptations to ensure protection is safe [28, 30, 31, 77]. Furthermore, clinical practice for suicidal patients is characterised by multiple conflicting goals which must be resolved by making trade-offs between higher- and lower-level goals [78], e.g., preventing immediate harm and working towards long-term health goals. Dynamic interactions and adaptations to ensure safe work are characteristics of a complex adaptive system [27, 28, 63, 64].

Relationships of trust in resilient practices for patients in suicidal crisis reflect that the complexity in clinical practice is characterised by the establishment of psychological and relational safety, which is only created through personalised and trusted relationships. Personalised relationships are the core characteristic of mental healthcare as a complex adaptive systems [31].

In a CAS there will be unpredictable consequences of standardised patient safety interventions due to the need for adaptations in clinical practice to ensure safe outcomes. For those designing patient safety interventions at the blunt end of the system, deviations from the procedure may be perceived as an error to be corrected [6]. However, while errors arise from approximate adjustments of procedures, it is also why everyday work in complex adaptive systems is safe [6]. To improve safety in complex adaptive systems, variability should be embraced, not erased [79]. This means that flexible and relationally based patient safety measures should be developed alongside the currently more standardised approaches. Protocols, procedures, and guidelines could for example form a starting point for reflections on how a certain practice might entail variability that go beyond these standardised measures and how the variability should be acted on.

The CAS perspective of clinical practice in suicide prevention also has implications for learning. To learn from variability, it is necessary to move from simple linear models (e.g., root cause analysis) to systemic models (e.g., resilience, CAS) focusing on why practices vary, succeed or fail at the clinical level, hospital management level and at the health system macro level [9, 30, 77].

### Supporting resilient practices 

This study describes resilient practices which outline complexity in the clinical work in suicidal inpatient care. Some generic lessons regarding how to approach patient safety and suicide prevention at the micro level of the system are drawn based on the RHC perspective.

In a CAS, it is impossible for the HCPs to anticipate all of the consequences of adjustments of procedures and trade-offs [80, 81]. Feedback systems are needed to acquire knowledge of how to adapt and its outcomes [82]. This implies that, systems in mental healthcare need to collect feedback on everyday clinical practice to learn what fosters success under various conditions (Safety-II), not merely from retrospective views of suicide incidents [6, 9, 14]. Suicidal patients provide valuable feedback on what makes them feel safe or unsafe, identify conditions for successful treatment, and those that may cause adverse events. All of this corresponds to findings in other domains of healthcare [83, 84]. Collecting patient feedback should be considered essential to support resilient practices for suicidal patients.

Furthermore, suicidal patients’ experiences should inform the development of patient safety measures. This study finding corresponds with the literature that identifies trust as fundamental for hospitalised patients’ feelings of safety [85, 86]. The relational component of patient safety is the most vital aspect of care from the patients’ perspective, and HCPs can strengthen the sense of safety in those patients by respecting them as human beings, validating their feelings and ensuring that they know that people care about them. A sense of safety is also linked to a sense of control. HCPs may strengthen patients’ sense of control by addressing underlying issues and mental illnesses during hospitalisation and adapting suicide risk assessments and therapeutic approaches to suicidal behavior to meet each patient’s needs. An intervention which may support resilient practices is the collaborative assessment and management of suicide risk (CAMS) that involves the exploration of the suicidal patients’ individual drivers, warnings signs along with addressing the patient’s pain and suffering [87, 88]. Furthermore, dialectical behavior therapy, which aims to improve patients’ emotional and practical coping skills is relevant [89, 90].

This study implies that HCPs apply sensemaking strategies to improve their situational awareness of suicidal behavior. These strategies are enacted to gain a fuller meaning of the information they have obtained and a sense of what is going on in complex and ill-defined situations [75, 76, 91]. Since obtaining feedback from the healthcare team is essential in the creation of situational awareness [74–76], training in suicide risk detection can benefit from multidisciplinary training involving HCPs who regularly interact as a team to establish a shared vision, values and mental models [92, 93]. HCPs need to be able to discuss their clinical judgement in everyday clinical practice with a team of colleagues [75, 76]. In dialectical behavior therapy, and evidence-based treatment for borderline personality disorder with a specific focus on suicidal behavior, discussion of clinical assessment with colleagues is an integral part of the intervention [89, 90]. Furthermore, the results indicate that opening a collaborative dialogue with the patient may also help HCPs’ sensemaking of suicidal behavior [87]. In this regard, sensemaking is concerned with supporting strategies that create a more comprehensible understanding that enables action [94]. Future studies are needed to develop complex interventions to improve HCPs’ shared situational awareness, non-verbal communication and relational skills in the patient contact, and may include the development of simulation training interventions for suicide risk assessment [95].

Following the literature, this study highlights the importance of having experienced HCPs [96], who are fully therapeutically engaged with the patient [97, 98] who balance the exertion of control and the building of the therapeutic relationship during observation [99]. Observation of patients at suicide risk is a resilient practice that entails watchfulness and sensitivity to cues of a patient’s mental state along with engaging with the patient and establish bonds of trust. The findings are in accordance with research depicting prevention of suicides in constant observation as intertwined with forming connections and regaining hope [97, 100, 101]. The finding supports that observation demands resilient strategies from the individual HCPs and required adaptations and the balancing of multiple goals in care [28, 30, 77, 102].

Lastly, this study implies that resilient practice should not rely solely on HCPs’ capacity to adapt without formal support systems. Without reliable sources of trust, the system is brittle. HCPs’ adaptive capacities may be overstretched and threaten the adaptive capacity of the system [103–105]. The findings also follow the literature finding that HCPs may distance themselves from suicidal patients’ emotions to protect themselves from emotional discomfort which may erode patients’ trust in their HCPs [106–108]. HCPs need to know they are supported, and thus they favour formal arenas for collegial trust, support [65, 109], supervision and training to ensure they can keep working with suicidal patients [66, 97]. Support systems are currently not considered a vital part of clinical guidelines for inpatient suicide prevention [10]. However, to support resilient practice, HCPs’ capacity to handle daily stress needs to be nurtured after incidents and on a daily basis. Clinical guidelines and patient safety policies related to inpatient suicide prevention need to include psychosocial support system and clinical supervision of HCPs.

### Strengths and limitations 

The ability to judge the quality of qualitative research rests on four characteristics: credibility, dependability, confirmability and transferability [110]. Triangulation of data sources (patients and professional groups), methods (focus groups, individual interviews and systematic review of literature) and the use of several researchers enhance the credibility of this synthesis: the confidence in the accuracy of the data and ensures that the research investigates what it intended to investigate. Credibility was strengthened by including a sample with sufficient information power [111], that covered significant variations and had relevant experiences with the phenomenon under study [112]. A sample size of 18 participants was considered adequate to ensure such information power when studying a heterogenic group of patients with suicidal behaviour [111]. A sample size of 18 and 25 HCPs was considered adequate to ensure variability across care settings (locked/open wards), diverse specialities (psychologists, nurses, medical doctors), gender, experience, expertise and patient diagnosis [111].

Dependability is strengthened in this synthesis by the provision of clear, detailed descriptions of all procedures and methods in the first author’s PhD thesis [45], the original studies [41–43], and by providing transparency through the published protocol [44], This allows for appraisal without the need to arrive at the same results [113].

Confirmability is the degree of neutrality and researcher bias [110]. Confirmability was strengthened through the sharing of the researchers’ backgrounds, preconceptions and pre-understandings to interpret the data [114]. This study adopted an inductive approach to analysis and curiosity about the experienced reality to describe resilient practices in this context, which reduces researcher bias through approaching data with sensitivity and openness [53].

Transferability is the extent to which the findings can be transferred to other settings, context or groups [113]. Qualitative data produce culturally situated knowledge which cannot be generalised to all inpatient practice settings [61]. The resilient practices described here are part of the processes of clinical practice, not linked to specific outcome measures (e.g., suicidal behaviour, symptoms). We therefore cannot draw conclusions on the effect of such practices related to suicidal behaviour and they should not replace current evidence-based system approaches to suicide prevention (e.g., [11–13, 22, 115–119]) However, the findings of this study can be conceptualised at a theoretical level and used to arrive at a deeper insight into the ontology of safety for suicidal patients [120]. This study focused on hospitalised patients who survived a suicidal crisis and resilient practices in mental health wards, as experienced by these patients and their HCPs. As such, the study conclusions do not pertain to patients dying from suicide or to patients who were not admitted to hospital wards during their suicidal crisis.

## Conclusion

Resilient practices for patients hospitalised with suicidal behaviour are experienced by those patients and their HCPs using three practice types: interactions capturing non-verbal cues, protection through dignity and watchfulness, and personalised approaches to alleviate emotional pressure. All of these practice types are grounded in relationships of trust.

These practices inform the conceptual development of resilience in inpatient suicide prevention. The prevention of inpatient suicide is characterised by complexity related to changing, interrelated and variable patient needs in clinical practice and to the unpredictability of suicidal threats. Thus, inpatient suicide prevention demands adaptations and high levels of sensitivity towards the patient. To improve patient safety in this context, variability in clinical practice must be embraced. It is important to acknowledge the uncertainty in clinical decision-making regarding suicide risk and to avoid concluding that HCPs have performed an unsuccessful suicide risk assessment as the root cause of patient suicides. It is not possible to eliminate all uncertainty of suicide risk at the individual patient level. Nevertheless, it is possible to reduce emotional demands placed on HCPs by acknowledging the complexity and challenges involved in clinical decision-making regarding suicide.

Strategies should strengthen feedback systems that foster communication between HCPs and between HCPs and their patients, and reinforce systems such as clinical supervision that ensure support for HCPs. This synthesis was limited to the suicide inpatient care at the micro level of the healthcare system. Future studies should include the roles of family, carers, and ward management.

## Data Availability

The datasets generated and/or analysed in this study are not publicly available due to ethical concerns and the sensitivity of the data, but non-identifiable data are available from the corresponding author on reasonable request.

## Abbreviations

HCP: Healthcare Professional
RHC: Resilient Healthcare
CAS: Complex Adaptive System
